# A rapid and accurate new bedside test to assess maximal liver function: a case report

**DOI:** 10.1186/1754-9493-7-11

**Published:** 2013-04-25

**Authors:** Sascha A Müller, Ignazio Tarantino, Marcello Corazza, Frank Pianka, Jürgen Fornaro, Ulrich Beutner, Cornelia Lüthi, Bruno M Schmied

**Affiliations:** 1 Department of Surgery, Kantonsspital St.Gallen, St.Gallen, Switzerland; 2 Institute of Radiology, Kantonsspital St.Gallen, St.Gallen, Switzerland; 3 Rorschacherstrasse 95, St.Gallen, 9007, Switzerland

**Keywords:** Liver function, Liver volumetry, Liver resection, Liver function measurement, LiMAx

## Abstract

**Background:**

In liver surgery, appropriate preoperative evaluation and preparation of the patient is of cardinal importance. The up-to-date, preoperative prediction of residual liver function has thus far been limited. As post-hepatectomy liver failure is a major cause of mortality, a new and simple bedside test (LiMAx) has been developed to predict postoperative liver function in conjunction with preoperative volumetric analysis of the liver.

**Case presentation:**

A 45-year-old patient presented with a cecal carcinoma and a large synchronous liver metastasis for major liver surgery. Liver function was determined by the LiMAx-test for the enzymatic capacity of cytochrome P450 1A2, which is ubiquitously and solely active in the liver. A solution of 2 mg/kg body weight ^13^C-labeled methacetin was injected as a bolus into an intravenous catheter and, thereafter, was metabolized into acetaminophen and ^13^CO2 and pulmonarily exhaled. The analysis of the ^13^CO2/^12^CO2 ratio was performed using online breath sampling over a period of maximally 60 minutes. Based on this test, a value of more than 315 μg/kg/h represents normal liver function. A laparoscopic right hemihepatectomy was planned during virtual resection with a residual liver volume of 48% and a preoperative anticipated residual LiMAx of 301 μg/kg/h. After successful resection, the initial postoperative LiMAx value was 316 μg/kg/h, indicating good liver function and a correct prediction of the outcome.

**Conclusion:**

In the presented patient, residual liver function could be accurately predicted preoperatively using a combination of the new LiMax test with CT-volumetry. This test might significantly improve preoperative evaluation and postoperative outcomes in liver surgery.

## Background

Improvements in surgical techniques, anesthetic protocols, and perioperative care have decreased morbidity and mortality and have contributed to an increase in extended liver resections. This more aggressive approach inevitably leads to smaller remnant livers with a substantially elevated risk of post-resectional liver failure (PLF) [1-3]. It was demonstrated that 80% of deaths caused by PLF occur after resecting more than 50% of the liver volume [4-6], and the PLF incidence increases with the number of resected segments [5]. Therefore, accurate preoperative volumetric calculation of total and partial liver volumes based on preoperative imaging and resection planning is crucial in the assessment of hepatic functional reserve and resectability, especially in cases of major liver resection and patients with underlying parenchymal disease [7]. Liver steatosis and steatohepatitis, for example, are associated with an increased risk of PLF after partial liver resection, especially after neoadjuvant chemotherapy, or in living donor liver transplantation [8]. Traditional methods estimating the hepatic functional reserve, such as liver enzyme tests and Child-Pugh classification, have clinical and practical limitations [9]. The major disadvantage of most quantitative liver function tests, such as the indocyanine green clearance test and the galactose elimination capacity, is that they are influenced by several factors such as hepatic blood flow and hyperbilirubinemia [10]. Moreover, no reliable cutoffs and guidelines could be defined. Consequently, clinical decisions mainly rely on the surgeon’s experience [1]. Until now no biological marker or score has been able to reliably measure total liver function or predict the postoperative outcome. Here, we present the LiMAx-test, which has been developed at the Department of General, Visceral and Transplantation Surgery at the Charité in Berlin [11]. The test has been successfully evaluated in many patients undergoing liver surgery and liver transplantation since 2003 [11,12]. This test is now used in all patients with planned liver resection in our surgical department and in several other European centers.

## The new LiMAx-test

This breath test is based on the hepatocyte-specific metabolism of the cytochrome P450 1A2 enzyme, which is active throughout the liver. After 3 hours of fasting, patients are placed in a resting horizontal position. The activity of the enzyme is measured by i.v. bolus injection of non-radioactive ^13^C-methacetin (2 mg/kg body weight). Ten minutes prior to the injection with ^13^C-methacetin, the baseline ^13^CO_2_/^12^CO_2_ ratio is recorded. ^13^C-methacetin (Euriso-top, Saint-Aubin, Cedex, France) is metabolized into acetaminophen and ^13^CO_2_, which is pulmonarily exhaled. The exhaled air is collected using a face mask (Humedics GmbH, Berlin Germany), and the ^13^CO_2_/^12^CO_2_ ratio in the air is determined using a modified nondispersive isotope-selective infrared spectroscopy (FANci2-db16, Fischer Analysen Instumente, Leipzig, Germany, Humedics GmbH, Berlin, Germany) [11,13]. Based on the ^13^CO_2_/^12^CO_2_ ratio and the body weight, the cytochrome P450 1A2 activity is determined using the so-called LiMAx value with the unit μg/kg/h (μg methacetin/kg body weight/hour). The analysis of the ^13^CO2/^12^CO2 ratio is performed over a period of 20 to 60 minutes. For normal liver function, a LiMAx value over 315 μg/kg/h is required. In healthy volunteers, the normal range of LiMAx was found to be 425 ± 67 μg/kg/h (range 311-575 μg/kg/h). Furthermore, the correlation between repeated measurements was found to be 0.82 [11].

## Case report

**Figure 1:**
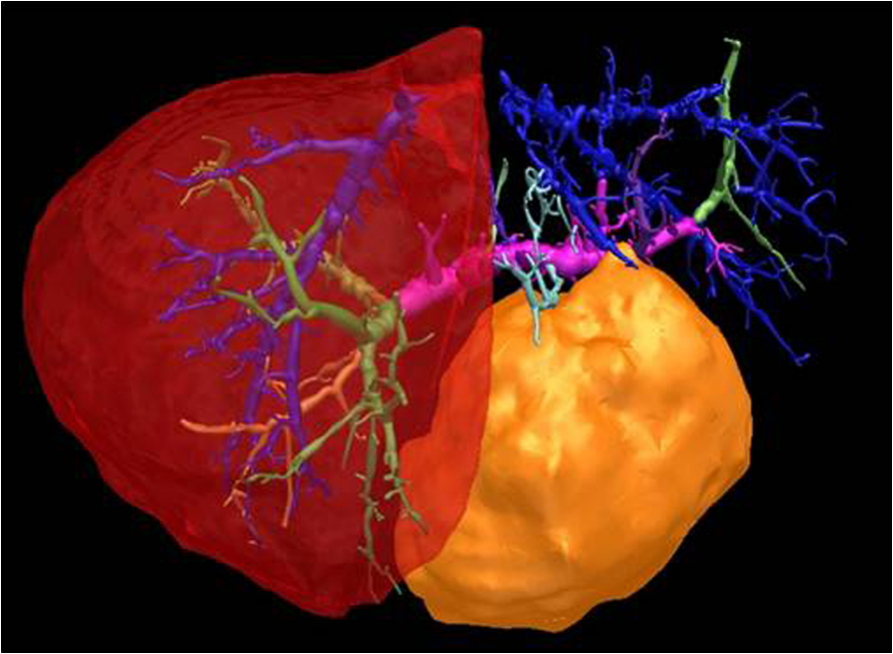
**Three-dimensional computation from a preoperative CT scan of the patient with a large liver metastasis (superior view).** The portal vein is pink and green, the hepatic veins are blue, and the tumor is yellow.

A 45-year-old patient in good general health presented with a cecal carcinoma and a large synchronous liver metastasis. After neo-adjuvant chemotherapeutic treatment with 5-FU, Irinotecan and Oxaliplatin, a “liver first” approach was decided by the tumor board. The preoperative LiMAx value was 622 μg/kg/h, functional liver volume was 1736 mL, and tumor volume was 351 mL. A laparoscopic left hemihepatectomy was planned during virtual resection with a functional resection of 895 mL, resulting in a residual liver volume of 841 mL (48%) and a pre-operatively anticipated residual LiMAx of 301 μg/kg/h (Figure 1). The laparoscopic left hemihepatectomy was performed under general anesthesia in the supine position using 5 ports according to the technique described by Pearce et al. [14]. Briefly after applying a nylon tape around the portal triad for the Pringle maneuver, the liver was completely mobilized. The resection plane was defined by intraoperative ultrasound. After vascular control, the dissection was performed by harmonic scalpel (Harmonic ACE; Ethicon Endo-Surgery, Cincinnati, OH, USA) and Cavitron Ultrasonic Surgical Aspirator (Valleylab, Boulder, CO, USA) under intermittent Pringle maneuver. After completion of adequate hemostasis, the specimen was removed in an impermeable bag (Endocatch; Ethicon Endo-Surgery) introduced through a 15-mm suprapubic port subsequently extended through a Pfannenstiel incision. The operation time was 5.5 hours, and the intraoperative blood loss was 300 mL.

After successful resection, the initial postoperative LiMAx value on postoperative day one was 316 μg/kg/h, indicating good liver function and correct prediction of the outcome. After an uneventful postoperative course, the patient was discharged on postoperative day six. Eight days later, an uneventful laparoscopic right hemicolectomy was performed to remove the primary tumor.

## Discussion

As impressively demonstrated by the presented case, the LiMAx-test in combination with a CT-based liver volumetry can reliably predict the postoperative liver function. Therefore, we adopted these preoperative tests as a standard procedure for every patient undergoing liver resection at our institution.

In 2010, Stockmann et al. published the LiMAx algorithm, which is an easily applicable clinical decision tree for preoperative resection planning [15]. This allows effective preoperative surgical risk evaluation to prevent major complications or even liver failure-related death. Normal liver function (LiMAx >315 μg/kg/h) allows the resection of up to four liver segments without further consideration. By contrast, patients with strongly impaired liver function (LiMAx <140 μg/kg/h, indicating significant hepatic injury) are not eligible for surgery as they are prone to developing postoperative liver failure even after minor liver resection. Patients with limited hepatic impairment and a preoperative LiMAx-value between 140–315 μg/kg/h are the most challenging ones. In these patients, precise liver volumetry is mandatory for a planned major liver resection (>four liver segments). Resections with an expected residual LiMAx value less than 80 μg/kg/h should be avoided because they are associated with a high mortality of up to 40%. Planned resections with a predicted residual LiMAx value between 80–100 μg/kg/h are considered critical (10.5% postoperative mortality), and thus alternative therapies or additional preoperative procedures should be considered (i.e., portal vein embolization, neo-adjuvant chemotherapy to downstage tumors). Liver resections with expected residual LiMAx values of >100 μg/kg/h can safely be performed.

The LiMAx test can clearly help to avoid post-resectional liver failure. Thus, one could expect a relevant impact on health care costs. Recently, Lock et al. published a study examining 45 patients after major liver resection [16]. Eight patients developed postoperative liver failure, seven of these died in the postoperative course. Patients developing post-resectional liver failure generated higher costs than patients without post-resectional liver failure (82,199 € [95%CI: 42,812 – 21,586 €] vs. 25,980 € [95%CI: 9,559 – 42,401 €]; p = 0.013) [14]. The proportion of intensive-care unit cost relative to total costs increased from 30% to more than 50%, and the cost for blood tests and products from the blood bank increased more than eightfold from 819 € [95%CI: 0 – 1,837 €] to 6,953 € [95%CI: 4,512 – 9,395 €]; p ≤ 0.001). In covariate analysis, overall costs related to post-resectional liver failure were 56,219 € [95%CI: 12,327 – 100,066 €]. Routinely applying the LiMAx test, we can achieve an optimized and individualized treatment for patients scheduled for liver resections. This ultimately leads to economic savings by significantly decreasing morbidity and mortality and the overall length of hospital stay.

## Conclusion

In the presented patient, residual liver function could be accurately predicted preoperatively by the combination of the new LiMax test with CT-volumetry. Considering the existing literature, the LiMAx test significantly improves preoperative evaluation and postoperative outcomes in liver surgery.

### Consent

Written informed consent was obtained from the patient for the publication of this case report.

## Competing interests

The authors declare no conflicts of interest. In particular, the authors do not have any specific conflict of interest related to the manufacturer of the LiMAx test, including intellectual property, patents or patents pending, industrial relation to the manufacturer, or speaker’s honoraria.

## Authors’ contributions

Data were prepared and reviewed by SAM, MC, CL and FP. JF reviewed radiological images. The manuscript was drafted by SAM, IT and UB and critically edited by BMS. All authors read and approved the final manuscript.
